# Flammability and Mechanical Testing of Sandwich Composite for Rolling Stock Structural Applications

**DOI:** 10.3390/ma17205125

**Published:** 2024-10-21

**Authors:** Marcin Kalinowski, Mirosław Szczepanik, Małgorzata Szymiczek

**Affiliations:** 1 Doctoral School, Silesian University of Technology, 2A Akademicka Street, 44-100 Gliwice, Poland; marcin.kalinowski213@gmail.com; 2 Alstom Konstal S.A., 9 Metalowców Street, 41-500 Chorzów, Poland; 3 Faculty of Mechanical Engineering, Silesian University of Technology, 18A Konarskiego Street, 44-100 Gliwice, Poland; malgorzata.szymiczek@polsl.pl

**Keywords:** rolling stock, composites, sandwich composites, static test, fatigue test, three-point bending test, finite element method (FEA), car-body shell (CBS)

## Abstract

Components made of composite materials are being increasingly used in the construction of rolling stock. Currently, the use of components made of composite materials as train structural elements is increasingly being considered. Non-structural components made of composites are most often found inside rail vehicles (e.g., the interior lining), while structural components made of sandwich composite materials can be used for the roof, sidewalls, and underframe constructions. This article provides a description of an innovative sandwich composite developed for a metro’s underframe, as well as the production process and preparation of the composite specimens. The main parts of the work are flammability and mechanical (static and fatigue) tests of the innovative sandwich composite. The scope of the flammability tests included the testing of the fire properties using the radial plate method, the optical density of smoke, and the content of toxic gases. The mechanical strength of the sandwich composite was examined during a flexural (three-point bending) test and a fatigue strength under a given dynamic load. The results presented in the article are very significant, both in terms of flammability and the mechanical strength tests. In order to produce large-size train components, appropriately large patches of component layers of the composite are required; this may pose production problems.

## 1. Introduction

The demand for research focused on the behavior of sandwich composites during static (three-point bending) and fatigue strength tests has arisen in recent years as a result of the growing acceptance of the composite construction concept in rolling stock production applications. In recent years, there has been an increase in interest in sandwich composites. Articles usually concern the application of sandwich composites in the design of load-bearing structures (e.g., the underframe) of rail vehicles [1].

In [2], the authors describe the application of an ant colony optimization (ACO) algorithm to the multiple-objective optimization of a rail vehicle floor sandwich panel. An optimization algorithm was used to search among material databases in order to identify structures of the sandwich composites that were optimal with respect to low mass and low cost. The result of the work was the identification of a broad range of sandwich composite solutions that provided savings of mass of up to 60% in comparison with existing solutions. It should be noted that a decrease in weight above 40% generated an increase in costs resulting from the price of individual components of the composite as well as the production process.

In [3], the authors draw attention to a multi-scale design approach of composite sandwich structures for the roof of a railway vehicle. The multistage procedure started with a cost analysis of materials and their manufacturing process and finished with an analysis of subcomponents and entire structures. The work presents the experimental campaigns that allowed the characterization of both the properties of the innovative sandwich composite and the bond’s behavior between its components. At the end, the authors discuss the results of a finite element analysis of the railway vehicle’s roof made of the proposed sandwich composite under loadings defined by European standards.

An equally important scientific work, although not directly related to the construction of rolling stock, is [4], an article on the behavior of composite multilayer beams under bending and compressive loads in order to assess the suitability of this construction system for sleepers for railway turnouts. The proposed innovative sandwich composite beam consisted of composite shells of glass fiber acting as face sheets and a phenolic core material. The mechanical properties of these beams were very similar to wooden sleepers for turnouts, which proves that composite sandwich structures have the potential to be used as replacement sleepers for railway turnouts.

An article [5] from a few years ago touches on the problem of the thermal resistance of layered composites for railway applications and describes a way of influencing improvements in the fire performance of sandwich composites without an effect on the internal composite structure. This effect is realized by using intumescent mats M10 and M20 with expansion ratios of 10:1 and 20:1, respectively, which are glued onto the heat load exposure area of the sandwich composite. When using mats, a significant reduction in the critical heat flux (CHF) and specific optical density of the smoke in the first 4 min of the test (VOF4) were observed for the sandwich composite in comparison with the test without them. Mat M20, due to its higher expansion ratio than mat M10 and excellent flame-retardant properties, could potentially be used in the production of rolling stock.

The material characterization presented in [6] focuses on the structural design of a composite-material front shield for a high-speed train. A sandwich structure was utilized, consisting of glass fiber epoxy face sheets and a polymeric foam core. Initially, the material properties and rate sensitivity of both the skin and core materials were assessed using a series of static and quasi-static tests. Subsequently, static and dynamic impact tests were conducted on the sandwich structure. The findings indicated that no significant strain-rate effects were observed across the tested materials under the variations of the test conditions examined in the study. The results demonstrated that the structural response of the sandwich was primarily influenced by the strength properties of the foam core material. Furthermore, the dynamic impact resistance of the sandwich structure was significantly enhanced by incorporating a network of resin walls within the foam.

Articles [7,8,9] contain an overview of the fundamentals, design, and manufacturing strategies used for sandwich composites. The structure, fabrication, and application-related challenges and probable future research directions, as well as the machining processes used for sandwich composites, are discussed in [7]. In [8,9], various techniques for the manufacturing of sandwich components intended for structural applications are summarized and discussed, focusing on the processing steps, the characteristics of both the techniques, and the resulting components, as well as examples of applications. The primary emphasis is on the most commercially prevalent manufacturing methods, although some lesser-known processing routes are also briefly addressed.

In the literature [10,11], one can find comprehensive information on composites such as material properties, manufacturing methods, and design aspects, as well as safety, maintenance, and inspection issues, while [11] focuses on fiber metal laminates.

**Figure 1:**
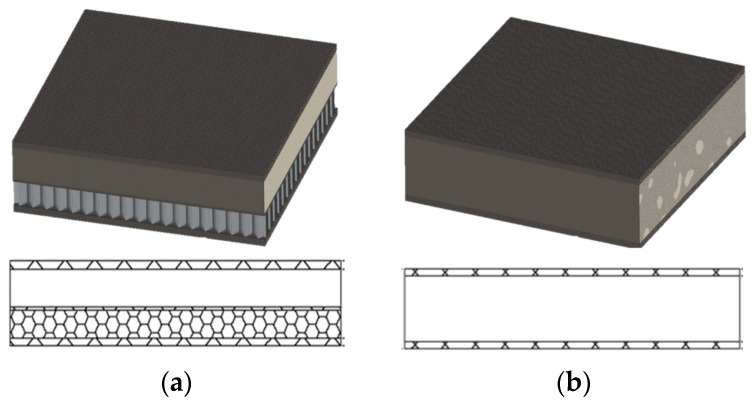
(**a**) A sandwich composite with a cellular core; and (**b**) a sandwich composite with a structural core.

A structural sandwich composite typically consists of two relatively thin surface layers, typically called “face sheets”, which are made of a stiff and relatively strong material, such as metal or fiber composite, bonded to a thick, lightweight material called a “core”. The concept of a structural sandwich composite is very similar to an I-beam, in which the flanges serve as the “face sheets” and the web as the “core”. The cores of structural composite materials can be divided into two main groups: cellular and structural. The cellular core consists of many identical “cells” containing an open space filled with air, enclosed by very thin walls, usually directed perpendicularly to the face sheets, e.g., metal honeycomb cores. The structural core is made of a continuous material formed in such a way that it separates the faces and becomes effective at transmitting shear forces [1]. Examples of such cores are all kinds of polymer foams and balsa wood (Figure 1).

Sandwich composite structures allow the optimization of the structure, mainly in terms of weight, like in the case of automotive body structures, aircraft parts or space structures, and sporting goods [12,13]. In the case of applications in rolling stock, a much greater advantage is the elimination of the welding process, due to its significant energy consumption and the need to employ qualified welders. In addition, the welding process is very difficult to control, which can result in many post-production errors that require repairs. Moreover, in addition to providing a very efficient load-bearing structure, the sandwich concept enables the design of multi-functional structures that combine load-bearing functions with thermal ones.

The disadvantage of sandwich composite materials, due to their complex structure, is their poor resistance to the resultant dynamic load during crashes and lower mechanical properties compared to isotropic metals, such as steel or aluminum alloys. In addition, they are difficult to recycle (due to their multi-material structure), and, for some cores in service, trapped moisture in their material causes corrosion problems.

### Structural Construction of Wagon (CBS) and Sandwich Composite Application

**Figure 2:**
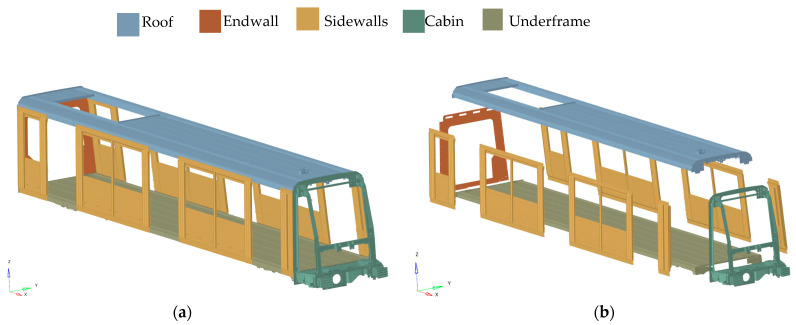
Components of a car-body shell: (**a**) general view; and (**b**) exploded view.

The structural construction of a wagon is called a car-body shell (CBS) and consists of five main components: the underframe, the roof, sidewalls, the endwall, and the cabin (only in the case of head cars). These components (example from metro’s CBS) are typically made of steel (spotwelded sheets with reinforcement profiles) or aluminum alloys (extruded profiles) and are presented in Figure 2. 

Sandwich composites can be used for the construction of all CBS main components except of the cabin, due to crash behavior requirements. The structures of the sidewalls, endwall, and roof made of sandwich composites must only meet the strength requirements that ensure the integrity of the CBS under the conditions provided in the EN 12663 [14] railway standard. The case is different when sandwich composites are used for underframe construction. In the case of this component, in addition to strength requirements (it is the most heavily loaded CBS component), it is also necessary to meet stringent flammability requirements.

**Figure 3:**
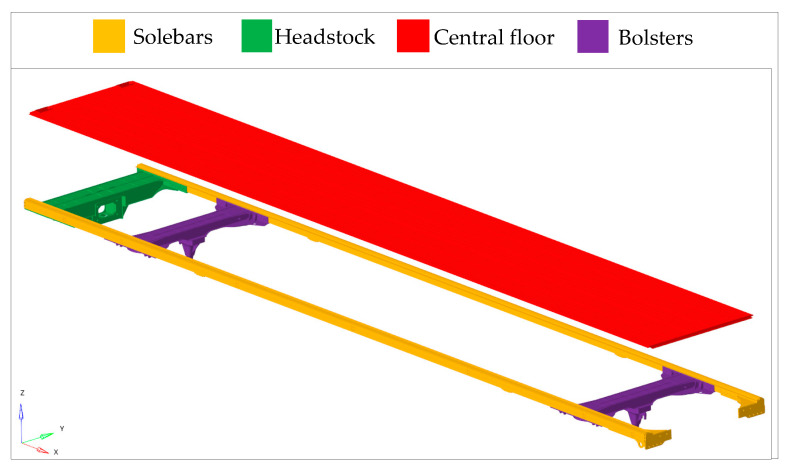
Exploded view of subassemblies of the underframe component.

In a modern metro design, the underframe (UF) is divided into four main subassemblies: the center floor, solebars (two per UF), bolsters (two per UF), and the headstock (Figure 3). Solebars (UF stringers) are the main load-bearing subassemblies in UF construction, which, together with cantrails (roof stringers), are responsible for maintaining the appropriate deflection arrow and the strength of the entire CBS from the static, fatigue, and crash points of view. Bolsters are the subassemblies responsible for the transfer of forces between the boogie and the CBS, and, as such, it is imperative that they are very resistant to loads. For these components, weight limitations are of little importance. The most important thing is the reliability of the structure. Headstocks transfer forces between wagons resulting from the train’s driving conditions (acceleration, braking, radius driving, and track unevenness), coupling, and, in extreme cases, also from collisions. Couplers or half-couplers are mounted to these subassemblies, which, in accordance with the railway standard EN 12663 [14], are loaded with a compressive force of 1500 kN and a tensile force of 1000 kN. The center floor is the least loaded subassembly belonging to the UF. It is responsible for transferring loads from passengers (standing and seated) and from equipment related to passengers (e.g., seats).

Taking into account the above functional description of the UF components, as well as advantages and disadvantages of sandwich composites, only the central floor is feasible to be made of sandwich composite materials instead of extruded aluminum profiles or reinforced steel sheets.

**Figure 4:**
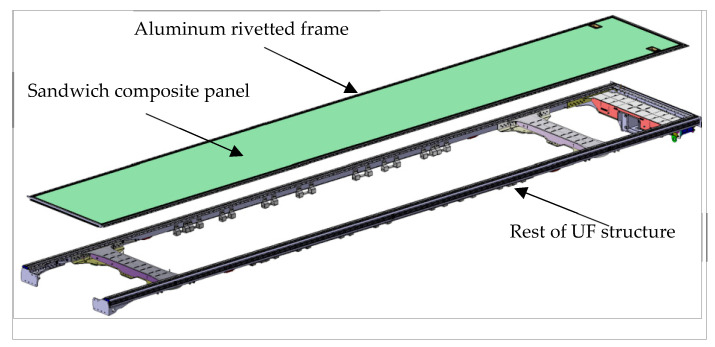
The method of mounting the central floor made of a sandwich composite onto the rest of the UF subassemblies.

The element connecting the sandwich composite center floor to the rest of the steel/aluminum alloy chassis subassemblies is the frame. The frame is connected to the composite by gluing. The connection between the frame and the rest of the UF subassemblies is carried out using high-strength structural (blind) rivets. The concept of composite central floor integration is shown in Figure 4.

The main aim of the work is to present the results of the physical (flammability) and mechanical tests of the proposed sandwich composite dedicated to the central floors of metro cars. The following tests have been performed:

(1) Flammability tests:
  Testing of fire properties using the radiant plate method;
  Smoke optical density test;
  Toxic gas content testing.
(2) Mechanical tests:
  Flexural test (three-point bending);
  Fatigue test.
  Additionally, the production process of the sandwich composite has been described.

## 2. Structure of Innovative Sandwich Composite

In order to propose a layered composite for a special metro car floor application, a review of materials that can be used in its structure was made. Next, the Delphi method (a method belonging to the group of heuristic methods in which knowledge, experience, and opinions of experts in a given field are used to make decisions) [15] for the generation of materials that could potentially be used for the construction of railway carriages was used. The following layers have been taken into account:

(1) Polymer foams (Armacell–polyethylene terephthalate and Airex-modified polyvinyl chloride);
(2) Aluminum or aramid honeycombs;
(3) Polymer composites with glass and carbon reinforcement.

The design criteria were to design a 61.5 mm-thick sandwich composite. This was due to the fact that the current solution used as the structural element of the underframe (extruded aluminum profiles) is of the same thickness. If the flammability, static, and fatigue strength tests are successful, the next stage will be to build a prototype of the entire chassis. At this stage, achieving the same thickness of the sandwich composite as the extruded aluminum panel will be a significant convenience, as it will not require the expensive adaptation of the rail vehicle components.

Manufacturability and cost aspects also had to be taken into account to build the special composite. Due to the adopted economic criterion (300 €/m^2^ of sandwich composite), honeycombs, despite their properties, were not taken into account in the following considerations. The same conclusion was reached in the case of carbon-reinforced composites. The manufacturing conditions of the CBS structure, such as the need to allow heavy tooling carts (such as welders, compressors, etc.) to be rolled over the upper surface of the center floor, necessitated the addition of an aluminum alloy (AW5754-T6) as a sandwich composite upper face sheet. The aluminum top layer is also resistant to cleaning agents and point loads.

It was assumed that foams and used resins should be approved for application in the railway industry in accordance with EN 45545 [16]—it was used as the second layer. 

The innovative sandwich composite structure is as follows:

- Aluminum sheet (thickness 2 mm);
- Adhesive interlayer—Primer SIKA 206;
- Glass fabric: Triaxal 557 g/m^2^ (0/45/−45)—10 layers (thickness 7 mm);
- Foam: Airex T90.100 (thickness 35 mm);
- Glass mat 300 g/m^2^—2 layers (thickness 0,5 mm);
- Foam: Airex T90.100 (thickness 10 mm);
- Glass fabric: Triaxal 557 g/m^2^ (0/45/−45)—10 layers (thickness 7 mm).

**Figure 5:**
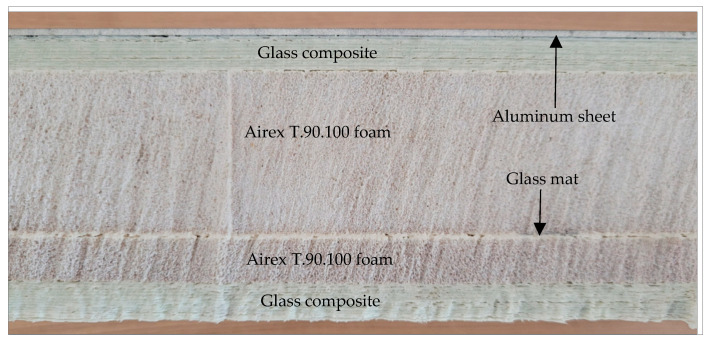
The innovative sandwich composite cross-section.

The matrix was POLIMAL 1608 resin. The panels were made with the technology described in the report quoted above. The weight of a 1 m^2^ hybrid panel was about 35.7 kg. The cross-section of the innovative sandwich composite is shown in Figure 5.

## 3. The Innovative Sandwich Composite Specimen Preparation

**Figure 6:**
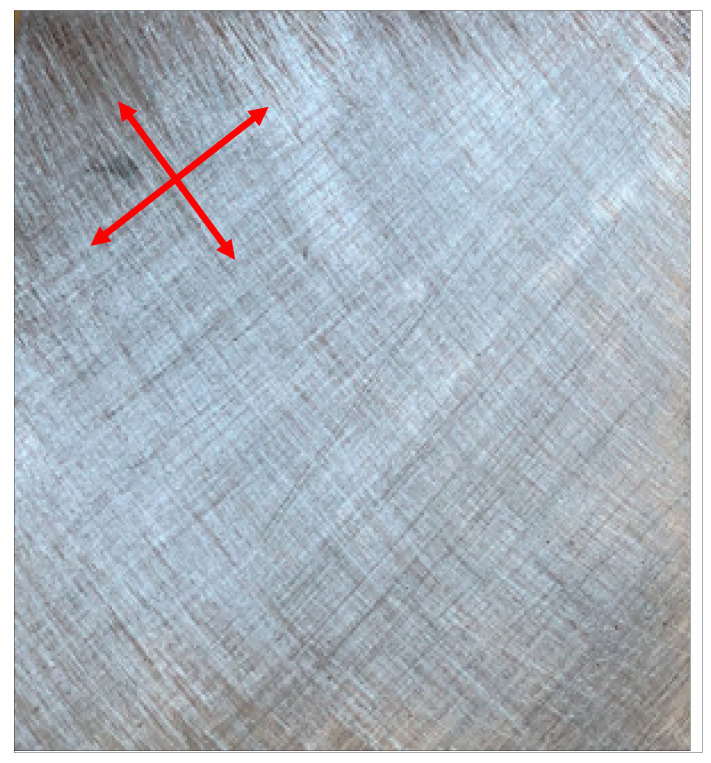
A prepared aluminum alloy sheet.

Hybrid floor panels were prepared by vacuum-assisted contact lamination. Firstly, aluminum sheets were prepared by roughening with P60 abrasive paper, as shown in Figure 6 (red arrows indicate the directions of grinding). This treatment is possible at the laboratory trials stage. This would be very time-consuming in the production process; thus, in serial production, aluminum sheets have to be bought pre-roughened.

**Figure 7:**
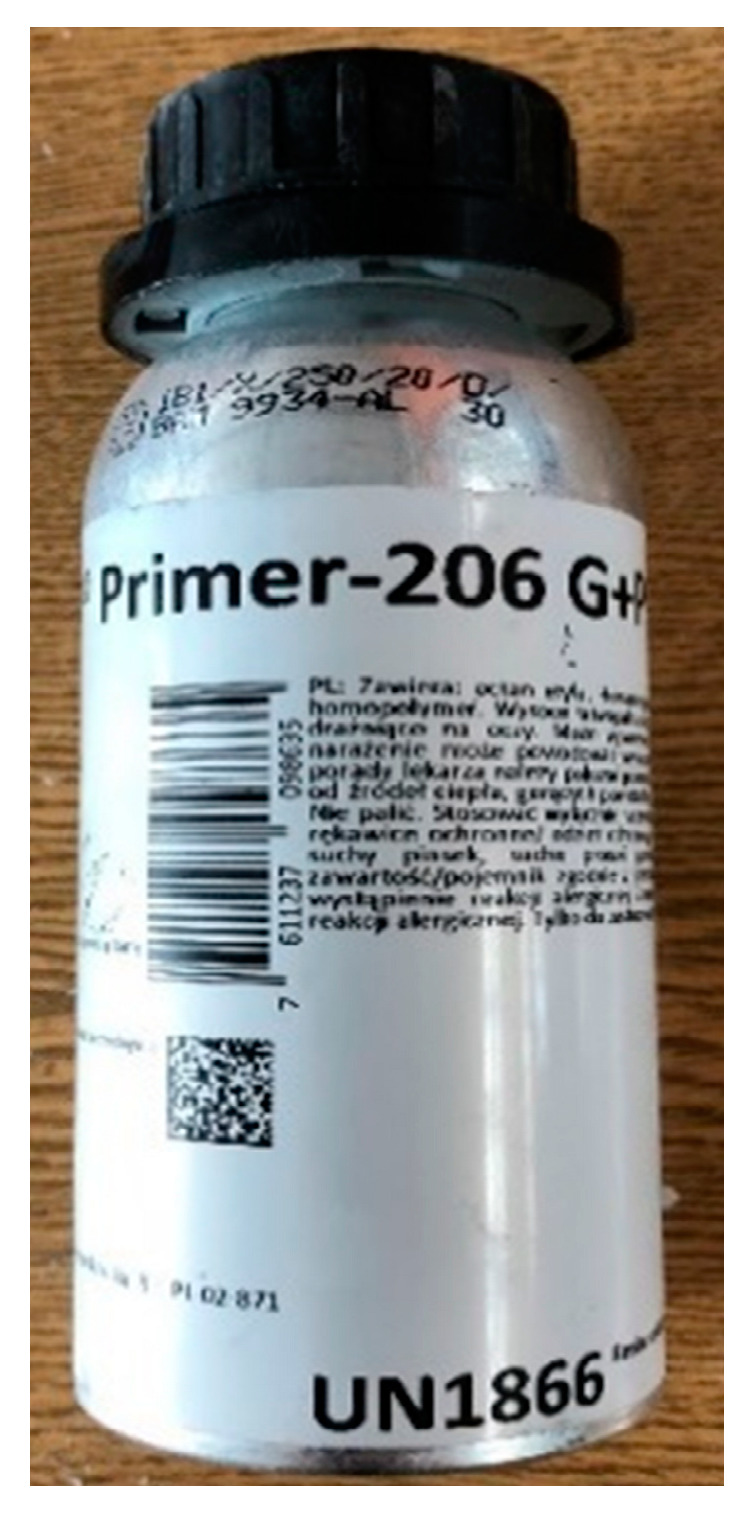
Primer SIKA 206.

In the next stage, the universal Primer SIKA 206 was manually applied to the matted and degreased sheet metal (Figure 7). In the production process, it is recommended to apply the primer automatically using rollers or automatic processes. The primer should be applied well in advance before further lamination to allow the solvent to evaporate.

**Figure 8:**
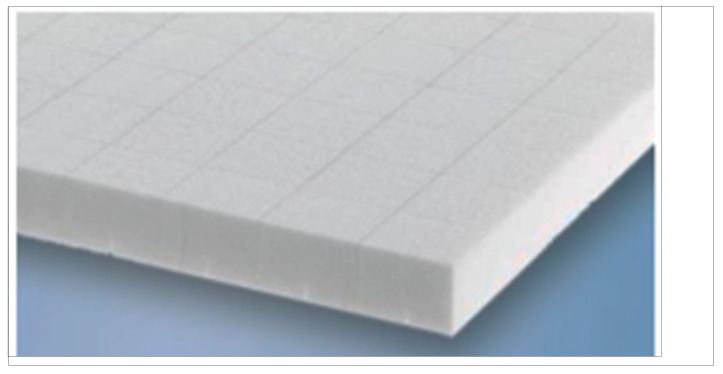
Airex T.90.100 foam with reinforcement in the longitudinal direction.

**Figure 9:**
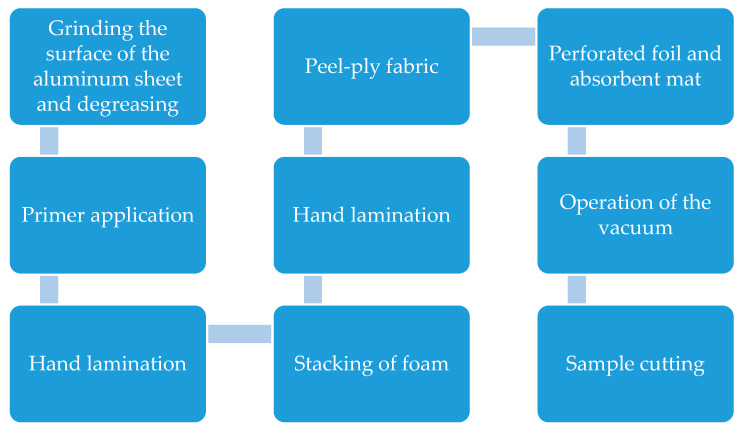
The production process of the specimens made with the innovative sandwich composite.

**Figure 10:**
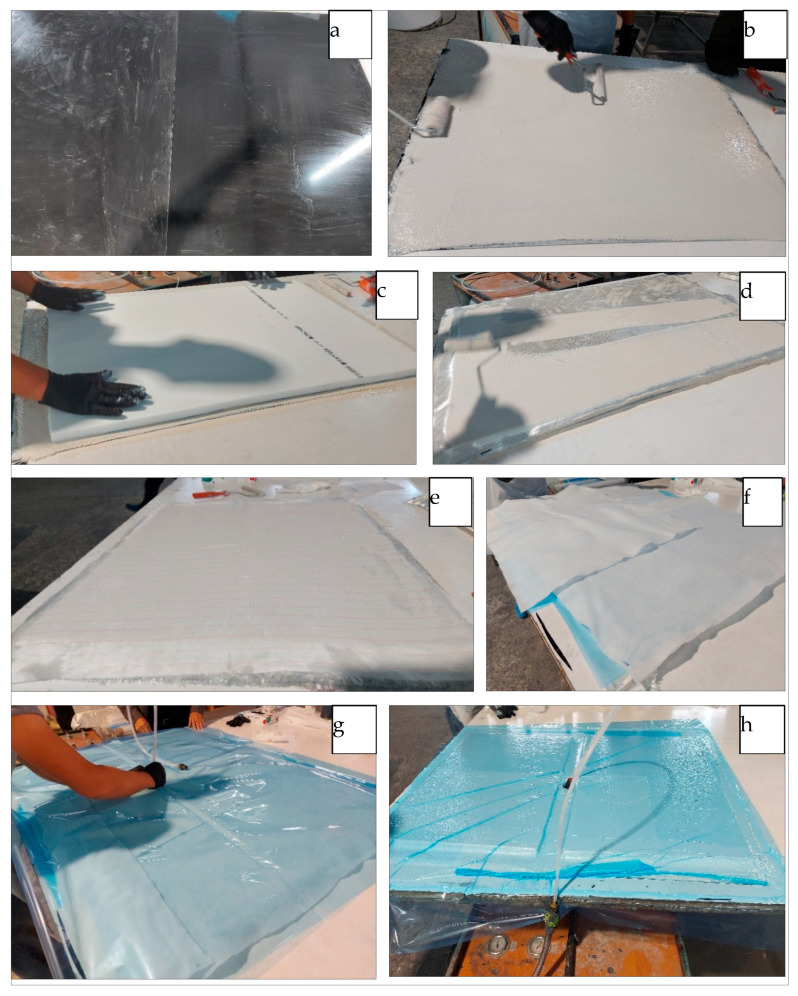
The production process of the specimens made with the innovative sandwich composite: (**a**) primer application; (**b**) hand lamination; (**c**) foam stacking; (**d**) hand lamination; (**e**) peel-ply fabric; (**f**) perforated foil and absorbent mat; (**g**) pressure plate installation and the vacuum bagging film; and (**h**) vacuum operation.

Flame-retardant polyester resin Polimal 1601 or Polimal 1608 was applied to the surface prepared in this way, which can be used in the railway industry on car bodies. Airex T.90.100 foam with a thickness of 35 + 10 mm was used as the core material (Figure 8). As can be seen, the foam is reinforced in the longitudinal direction, which is important for its operational properties. The production process of the specimens made with the innovative sandwich composite is presented in Figure 9 and Figure 10. 

The two-component epoxy adhesive Loctite 9466 (adhesive with a long open time) was used for the bonding process. This adhesive is characterized by high peel and shear strength and resistance to the effects of solvents.

It should be noted that, in this type of application, it is important to carry out a full range of tests, also aging in the thermal shocks, which will allow the verification of the connection properties in the long term. There are many adhesives on the market that can potentially be used in this type of application. At this stage of the work, only a few selected adhesives were tested, of which only Loctite EA 9466 provided the right bond.

## 4. Flammability Tests of the Innovative Sandwich Composite

### 4.1. Tested Samples

The samples for flammability tests were made by lamination technology with vacuum. The composite structure consisted of the following:

- Aluminum sheet;
- Adhesive interlayer—Primer SIKA 206;
- Glass fabric: Triaxal 557 g/m^2^ (0/45/−45)—5 layers;
- Foam: Airex T90.100;
- Glass fabric: Triaxal 557 g/m^2^ (0/45/−45—2 layers.

The matrix was POLIMAL 1608 resin (Table 1).

### 4.2. Testing of Fire Properties Using the Radiant Plate Method

**Figure 11:**
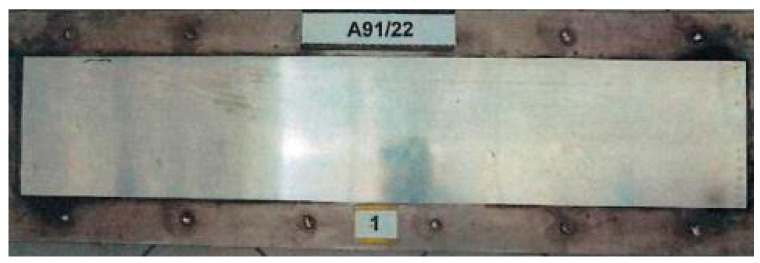
The sample for testing of fire properties using the radiant plate method.

Tests of fire properties using the radiant plate method in adherence with the EN ISO 9239−1standard [23] were carried out on six samples with dimensions (1050 ± 5) mm × (230 ± 5) mm, three along the direction of rolling aluminum sheets, and three in the perpendicular direction. The view of the prepared test samples is shown in Figure 11.

The method consists of the impact of a gas-fed radiating plate inclined at an angle of 30 degrees on a sample located in the horizontal position below the plate. The sample is exposed to a specific heat flux. The flame acts on the hotter end of the sample.

### 4.3. Smoke Optical Density Tests

**Figure 12:**
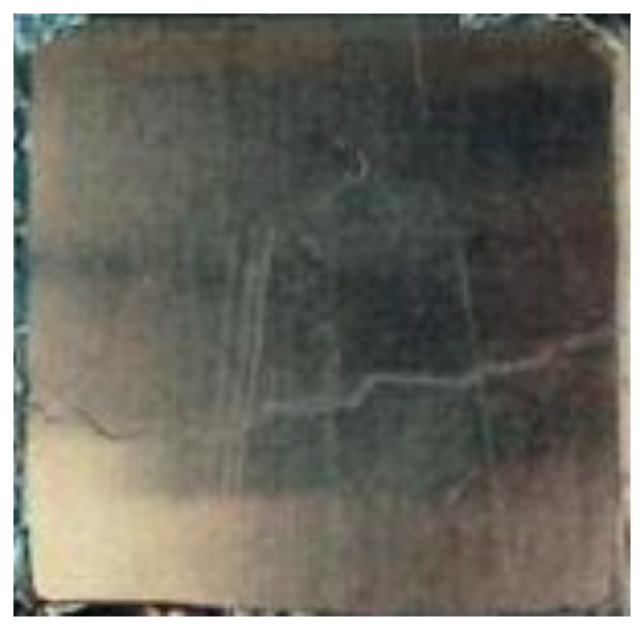
The sample for smoke optical density testing.

Smoke optical density tests were carried out in accordance with EN ISO 5659-2 [24] on six samples with dimensions of 75 × 75 mm. Before the tests, the samples were conditioned at temperature 23 ± 1 °C; humidity 50 ± 2.1%; and time 94 h. The view of the test sample is shown in Figure 12.

**Figure 13:**
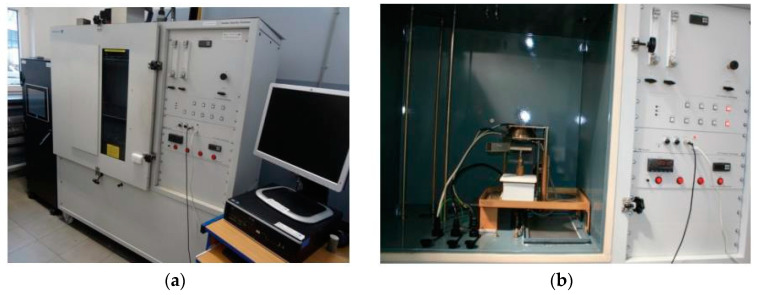
(**a**) Station for determining the optical density of smoke using the single chamber; and (**b**) interior of the test chamber [25].

Smoke optical density tests according to EN ISO 5659-2 [24] consisted of measuring smoke generation from the exposed surface of a polymer hybrid composite sample. Samples not exceeding 25 mm in thickness were placed in the horizontal position and subjected to a specific level of thermal radiation in a closed chamber with or without a pilot flame. Figure 13 shows the stand for determining the optical density of smoke. The tests were carried out at a temperature of 25.6 ± 2 °C, humidity of 43.1 ± 1%, and critical flow factor (Cf) = 5.19. The heat flux was 25 kW/m^2^ with a pilot flame. The samples were mounted without a wire mesh.

### 4.4. Toxic Gas Content Testing

The tests were carried out by EN 17084 [26] on the samples described in the smoke optical density tests. The samples were conditioned under the same conditions. Tests were also carried out in the chamber shown in Figure 13. To assess the toxic products that are formed during the combustion of gases in the smoke chamber, the standard toxicity index (CIT_G_—Conventional Index of Toxicity) was used. It was calculated on the basis of the test results. The concentrations of the following eight gases were analyzed: carbon dioxide (IV) CO_2_, carbon monoxide (II) CO, hydrogen bromide HBr, hydrogen chloride HCl, hydrogen cyanide HCN, hydrogen fluoride HF, nitric oxide NO_2_, nitric oxide (II) NO, and sulfur oxide (IV) SO_2_. The standard CIT_G_ toxicity index is determined by the formula:

(1) $$CIT_{G}=0.0805\overset{i=8}{\underset{i=1}{\sum }}\frac{c_{i}}{C_{i}}$$

where:

$c_{i}$—concentration of the i^th^ gas, measured in mg/m^3^ in the smoke chamber by PN-EN ISO 5659−2;
$C_{i}$—reference concentration of the i^th^ gas, measured in mg/m^3^. Reference concentrations are given in Table 2.

The factor 0.0805 is determined by the following quantities:

- The burning of 0.1 m^2^ of the exposed product;
- Gaseous volatile combustion product dispersion in 150 m^3^;
- A volume of the test chamber of 0.51 m^3^;
- Exposed surface area of the tested sample of 0.004225 m^2^.

The reference values are based on the IDLH [Immediately Dangerous to Life and Health] values recognized by NIOSH (National Institute for Occupational Safety and Health) as limits for a person’s exposure to gaseous components. The gases were collected in the fourth and eighth minutes of the test during the measurement of smoke optical density emission in the smoke chamber according to EN ISO 5659-2 [24].

## 5. Mechanical Tests of the Innovative Sandwich Composite

### 5.1. Flexural Test

**Figure 14:**
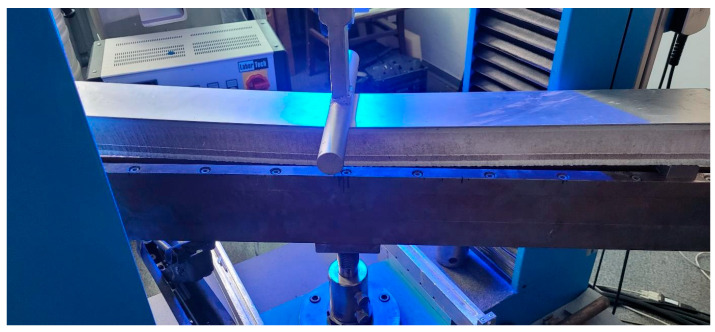
The sample during the three-point bending test.

Flexural strength tests were carried out on samples with dimensions of 100 mm × 1200 mm × 60 ± 2 mm. The samples were wet-cut on a diamond saw. Before the tests, the samples were conditioned in a thermal chamber at 60 °C and 50% humidity. Flexural strength tests were carried out on the LabTest 6.100 machine (Labortech s.r.o, Opava, Czech Republic) equipped with the MERCURY extensometer. The support span was 960 mm (16 times the overall thickness). The test speed was 5 mm/min. The test was carried out according to the ISO 178 [19] standard. Figure 14 shows the sample during testing.

### 5.2. Fatigue Test

**Figure 15:**
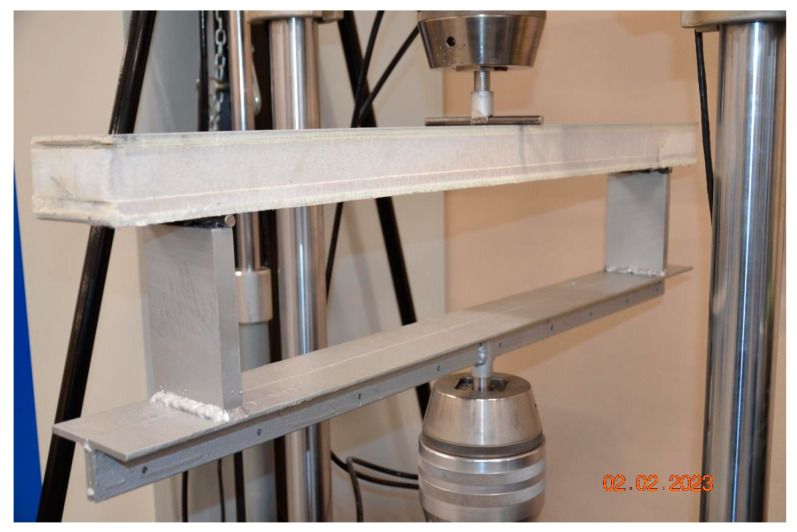
The measuring system scheme.

Fatigue tests were carried out on a machine providing a cyclical load in the assumed range by MTS. The spacing of supports was 960 mm. The supports were half-cylinders with a radius of 10 mm. In order to carry out the tests, instrumentation was made to ensure the attachment of samples at the length of 1100 mm and thickness of 60 mm. Figure 15 shows how the sample was loaded. The samples were always loaded from the side of the aluminum sheet. The test was carried out according to the ISO 178 [19] standard and internal company requirements. The tests were carried out with the following maximum loads:

- 480 N;
- 1000 N;
- 2000 N.

**Figure 16:**
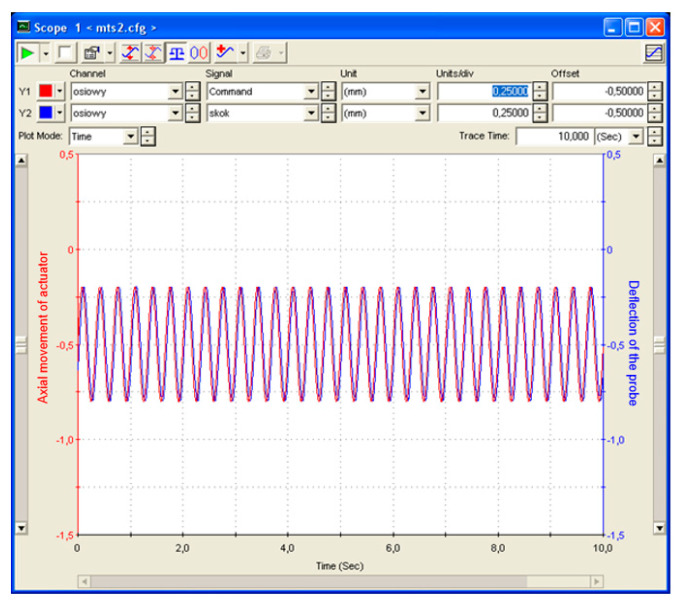
Deflection amplitude during fatigue test.

The fatigue test force of 480 N results from the design assumptions for metro cars, for which a load of six passengers (each weighing 80 kg) on an area of 1 m^2^ is assumed. Considering that the tested sample has a 10-times-smaller surface, the fatigue test force equal to 480 N is obtained. Loads of 1000 N and 2000 N were selected based on the results from the three-point bending test and the result from the maximum loads determining the work of the tested samples in the elastic range. Three samples were tested for each range. A frequency of 3 Hz was assumed. The minimum number of cycles was 1,000,000. Figure 16 presents the deflection amplitude during the fatigue test.

## 6. Test Results

### 6.1. Flammability Tests of the Innovative Sandwich Composite

#### 6.1.1. Testing of Fire Properties Using the Radiant Plate Method

No ignition of the samples was observed during the tests. The results of the study are presented in Table 3.

**Figure 17:**
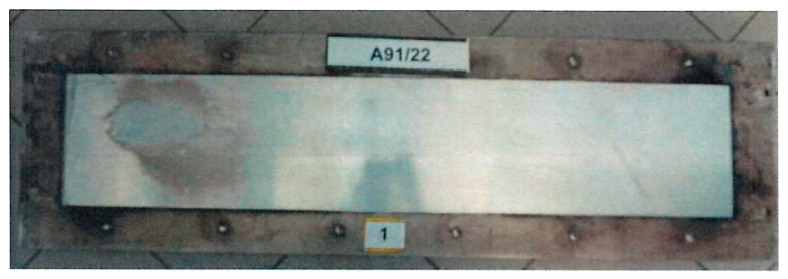
The sample after the testing of fire properties using the radiant plate method.

The sandwich composite panel meets the requirements of EN 45545-2:2020+A1:2023 [27] standard for R10 in terms of CHF at the HL1, HL2, and HL3 hazard levels. Figure 17 presents the sample after the fire properties testing using the radiant plate method.

#### 6.1.2. Smoke Optical Density Tests

The results of the tests carried out are presented in Table 4. None of the samples caught fire.

The smoke optical density tests carried out indicate that the proposed structure meets the requirements of the EN 45545-2:2020+A1:2023 [27] standard for R10 in terms of smoke optical density at the HL1, HL2, and HL3 hazard levels.

#### 6.1.3. Toxic Gas Content Testing

The results of the toxic gas content are presented in Table 5.

The result meets the requirements of EN 45545-2:2020+A1:2023 [27] standard in the field of CITG for RIO at the HLI, HL2, and HL3 risk levels. During the tests, no ignition of the samples was observed.

### 6.2. Mechanical Tests of the Innovative Sandwich Composite

#### 6.2.1. Flexural Test

**Figure 18:**
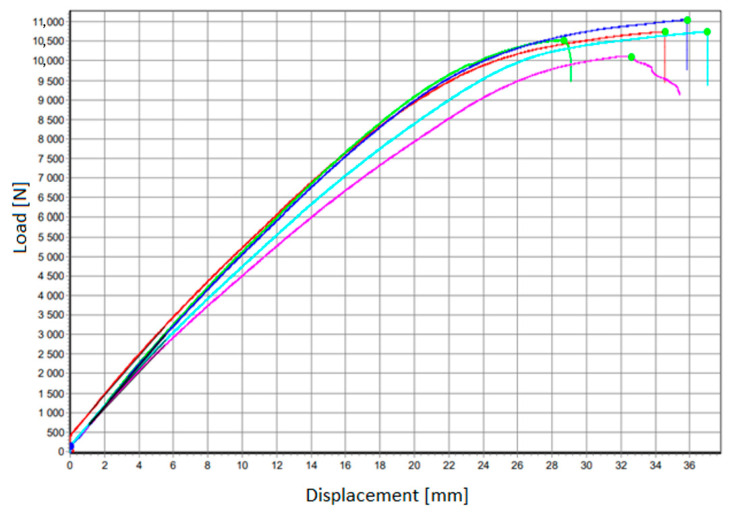
Load–displacement curve.

Figure 18 presents the load–displacement curve for all tested samples. The flexural strength was calculated from the formula:

(2) $$\sigma =\frac{3\cdot F\cdot l}{2\cdot b\cdot h^{2}}$$

where:

σ—flexural strength [MPa];
F—load [N];
L—supports span [mm];
b—width of sample [mm];
h—thickness of sample [mm].

**Figure 19:**
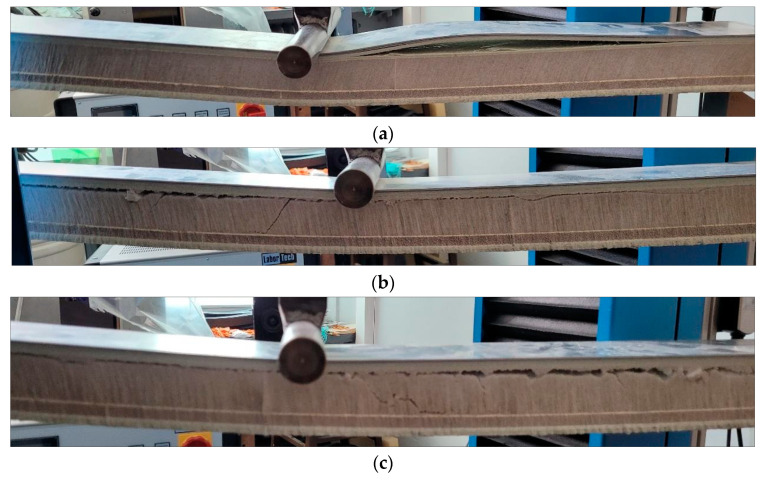
The sandwich composite damage: (**a**) delamination of the laminate; force = 10,100 N; purple line on the graph; (**b**) shearing of the foam–laminate adhesive layer with foam cracking; force = 10,600 N; red line on the graph; and (**c**) cracking of the laminate and foam; force = 11,000 N; blue line on the graph.

The average flexural strength was 39 MPa, with a standard deviation = 1.4. All samples show a similar character of the curves. Exemplary images of sample damage cut out of the panels are shown in Figure 19.

The force under which the layered composite was destroyed was in the range of 10−11.3 kN. As a reference, in the case of the three-point bending test, a static test force of 800 N can be used, which corresponds to the load of ten passengers (each weighing 80 kg) on an area of 1 m^2^ (assumption for dimensioning the central floor for exceptional payload). Considering that the tested sample is 1/10th the surface, the reference static test force equal to 800 N is obtained. As it was demonstrated, the innovative sandwich composite withstands a load at least 12.5 times higher than required.

The flexural strength of hybrid composite panels is 39 MPa. The observed failures correspond to foam shearing and cracking into the foam bonding mat layer. No failure was observed on the side of the tensile fibers—the opposite side from the load. The foam used absorbs the loads exerted to some extent.

#### 6.2.2. Fatigue Test

**Figure 20:**
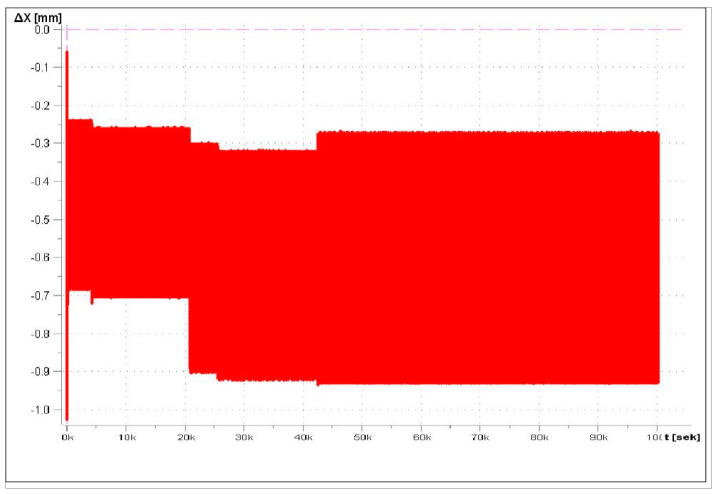
Change in strain in function of time for 0−480 N.

**Figure 21:**
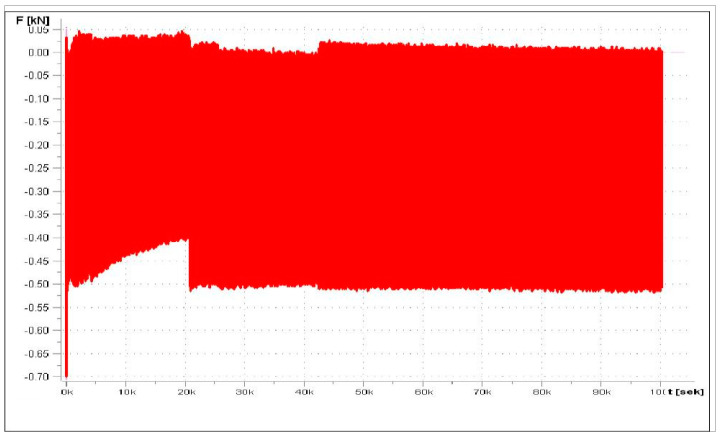
Change load of time for 0−480 N.

Figure 20 shows the dependence of deflection as a function of time for 480 N, and Figure 21 presents the load-versus-time dependency. The attempt was aborted at 1,114,156 cycles.

The maximum displacement for the 480 N load was 0.32 mm, while, for 1 kN, it was 0.9 mm, and, for 2 kN, it was 1.67 mm. Potential damage of the samples on the cross-section was verified visually. The damage was observed in the tested samples. The foam absorbs part of the load.

The fatigue tests carried out in the assumed conditions did not damage the structure of the tested material. For 480 N, the deformation is 0.32 mm and complies with the assumed requirements.

## 7. Manufacturing Process of Innovative Sandwich Composite

The proposed structures of sandwich composite panels can be manufactured using two methods:

- Joining smaller floor panels together;
- Manufacturing the entire floor.

In the case of the first solution, manufacturing the floor in sections of 2.9 m, assuming that the width of the carriage is 2.76 m, is suggested. Each floor will consist of four panels joined on a hinge. The sleeves will be laminated into the composite panels in order to insert the rod. These panels will be made using the RTM method (resin transfer molding) with vacuum support and compression with the assumed pressure.

In the second solution, the floors will be manufactured as one part. This is possible by modifying the known pultrusion technology for the production of continuous composite sections and profiles. The fabrics will be fed from spools as roving. However, the feeding devices must be able to perform a reciprocating movement in addition to the rotary movement so that the joining lines of the individual strands do not occur in the same place. The resin, apart from the external supersaturation (as in pultrusion), will be sprayed between the individual layers. Foam sheets will be inserted between the cladding, and the entire structure, composite–foam–composite, will be placed on aluminum sheets with the dimensions of the entire floor. The sheet will be fed with transporting devices.

In both solutions, the aluminum sheet, which is an integral part of the composite, must be blasted and then primed to ensure proper adhesion between the aluminum sheet and the composite.

Joining smaller floor panels together has a fundamental advantage—flexibility in terms of production by sub-suppliers. Smaller panels can be manufactured simultaneously by smaller companies, which will significantly affect the production time and probably also price. On the other hand, integrating smaller panels into the entire chassis will be more time-consuming.

Manufacturing the entire underframe, in turn, will reduce the time needed for integration to zero. However, the production of such a large component will be difficult and complex, which will limit the number of sub-suppliers capable of undertaking such a task, which will negatively affect the production time and its costs.

## 8. Conclusions

The method for manufacturing the innovative sandwich composite presented in the article has its limitations, the most important of which concerns the influence of the arrangement of reinforcing materials on the operational properties. It is mainly related to the width of available fabrics, which results from the possibilities of the weaving mill. The proposed solutions require the modification of existing technologies, which is related to research and technological trials.

The flammability tests of the innovative sandwich composite were successful. It was proven that this composite meets the requirements of fire properties tests using the radiant plate method, smoke optical density, and toxic gas content.

The mechanical strength tests were also successful, confirming the sufficient strength of the layered composite in the three-point bending test (safety factor equal to 12.5) and in the fatigue strength test (no damage to the composite or permanent plastic deformation after 1,114,156 cycles with a one-sided pulsating load of 480 N).

The positive results of flammability and mechanical strength tests also proved that it is possible to produce a layered composite that is able to combine the functions of a load-bearing component with a fire barrier. This will allow us to avoid adding special components constituting a thermal barrier in the construction of metro vehicles, which will have a very positive impact on the economic aspect (fire barrier components are expensive) and production (shortening of production time).

The next stage of the research may be the verification of the acoustic properties of the solution and the optimization of the arrangement of layers in order to reduce the costs and weight while maintaining satisfactory flammability and mechanical strength properties.

Future work will concern the intelligent optimization of the composite in terms of the selection of the number, type, and thickness of the layers [28,29,30].

## Figures and Tables

**Table 1:** Properties of polyester POLIMAL 1608 resin.

Properties	Standard	POLIMAL 1608
Viscosity 25 °C [mPa·s]	ISO 3219-2 [17]	500–800
Gel time [min]	DIN 16945 [18]	10–20
Flexural strength [MPa]	ISO 178 [19]	60
Tensile strength [MPa]	ISO 527 [20]	40
Strain at break [%]	ISO 527 [20]	0.8
Heat deflection temperature (HDT) [°C]	ISO 75 [21]	100
Barcol hardness [°B]	ASTM D 2583 [22]	55

**Table 2:** Reference concentrations of gaseous components [25].

Gas Component	Reference Concentration [mg/m^3^]
CO_2_	72,000
CO	1380
HF	99
HCl	75
HBr	55
HCN	25
SO_2_	38
NO_X_	262

**Table 3:** Results of fire properties tests using the radiant plate method.

Symbol	Parameter	Samples			Measurement Uncertainty at the Confidence Level 95%, k = 2	Test Results
		1	2	3		
CHF	Critical Heat Flux [kW/m^2^]	11.5	11.5	11.5	6.10%	11.5 ± 0.7

**Table 4:** Smoke optical density results.

Symbol	Parameter	Samples			Mean
		1	2	3	
m_p_	Initial weight [g]	82	81.5	85.1	82.9
m_k_	Final weight [g]	82	81.2	85	82.7
σ_m_	Loss of weight [g]	0	0.3	0.1	0.1
g	Sample thickness [mm]	25	25	25	25
t_c_	Ignition tome [s]	-	-		
t_k_	Extinguish time [s]	-	-		
t	Test time [s]	600	600	600	600
D_c_	Optical density of a pure beam	0.45	0.39	0.35	0.4
D_s max_	Maximum optical density of the smoke	0.62	0.48	0.56	0.55
D_s_ (4)	Optical density of the smoke after 4 min	0.29	0.31	0.41	0.34
VOF_4_	Total value of the specific optical density of the smoke in the first 4 min of the test	0.98	0.78	1.05	0.94

**Table 5:** Standard CITG toxicity index at 4 and 8 min of testing.

Parameter	Samples			Measurement Uncertainty at the Confidence Level 95%	Test Results
	A91.7/22	A91.8/22	A91.9/22		
CIT G(4)	0.009	0.005	0.007	±8.7%	0.007 ± 0.001
CIT G(8)	0.016	0.009	0.013		0.013 ± 0.001

## Data Availability

All data generated or analyzed during this study are included in the present article.
